# Scale‐Dependent Biodiversity Responses to Drying and Land Use in River Metacommunities Facing Global Change

**DOI:** 10.1111/gcb.71094

**Published:** 2026-09-04

**Authors:** Pedro Paulo Souza‐Lopes, Thibault Datry, Carla Ferreira Rezende, Andros T. Gianuca

**Affiliations:** ^1^ Graduate Program in Ecology Federal University of Rio Grande Do Norte Natal Rio Grande do Norte Brazil; ^2^ INRAE, UR Riverly, Centre de Lyon Villeurbanne France; ^3^ Postgraduate Program in Ecology and Natural Resources, Department of Biology Federal University of Ceará Fortaleza Ceará Brazil; ^4^ Department of Ecology Federal University of Rio Grande Do Norte Natal Rio Grande do Norte Brazil

**Keywords:** beta diversity, biodiversity scaling, dispersal limitation, environmental filtering, freshwater biodiversity, intraspecific aggregation, species abundance distribution

## Abstract

Global change is increasing river drying and transforming surrounding landscapes through human land use, with important consequences for freshwater biodiversity. Yet how river drying and land use jointly influence biodiversity across spatial scales remains poorly understood. Using macroinvertebrate data from 49 riverine metacommunities in southeastern France, we examined how river drying and land use affect biodiversity across spatial scales and the metacommunity properties underlying these patterns. We estimated species‐area relationship (SAR), with intercept and slope of SAR curve representing patterns analogous to alpha‐ and beta‐diversity, respectively, and fitted structural equation models to assess how flow intermittence, land use (% of non‐natural surface), and network size affected the metacommunity properties underlying these patterns, including intraspecific aggregation, species abundance distribution, total abundance, and regional species pool. Flow intermittence increased intraspecific aggregation and reduced evenness, leading to a lower SAR intercept and a steeper slope, reflecting lower local diversity but greater compositional differentiation among non‐perennial rivers, and revealing strong scale‐dependent responses of biodiversity to drying. Trait analyses further showed that flow intermittence favored aerial passive dispersers while reducing the relative abundance of aquatic active dispersers, suggesting that altered dispersal dynamics in non‐perennial river networks contribute to biodiversity organization. In addition, land use primarily influenced biodiversity through nonlinear effects on the metacommunity species pool, with intermediate levels of land use associated with the highest regional diversity. Moreover, land use weakened the positive effect of flow intermittence on aggregation, indicating that anthropogenic pressures can homogenize metacommunities in drying regimes. Our findings demonstrate that flow intermittence strongly influences biodiversity across spatial scales through different mechanisms rather than uniformly reducing it. As climate change is forecast to increase the proportion of non‐perennial rivers worldwide, our results highlight the importance of incorporating metacommunity processes and multi‐scale diversity patterns into the conservation and management of these ecosystems.

## Graphical abstract

Our analysis of riverine metacommunities reveals that river drying and land use interact to restructure communities by altering species abundance, aggregation, and regional pools, producing scale‐dependent effects on biodiversity. These findings highlight the need for tailored management strategies for the effective conservation of both perennial and non‐perennial river networks.

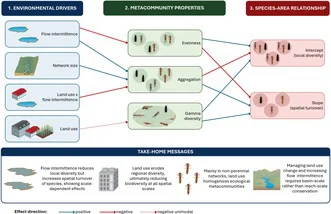

## 1 Introduction

The ongoing conversion of natural habitats into urbanized and agricultural landscapes is impacting biodiversity and ecosystem processes at multiple spatial scales (Barnosky et al. 2011; Foley et al. 2005; Habibullah et al. 2022; Yang et al. 2021). Such impacts are particularly severe in freshwater ecosystems, which contribute disproportionately to Earth's biodiversity, despite their relatively small area (Balian et al. 2008; Strayer and Dudgeon 2010). Indeed, there is evidence that freshwater organisms are declining at faster rates than their terrestrial and marine counterparts (Collen et al. 2009; Dudgeon et al. 2006). In river ecosystems, anthropogenic impacts (e.g., water abstraction and diversion) combined with climate change are increasing the extent of non‐perennial reaches, sections of rivers that dry or cease flowing each year, causing losses for ecosystem functioning and stability across spatial scales (Escobar‐Camacho et al. 2025; Gianuca et al. 2024).

Non‐perennial river reaches are prone to substantial diel fluctuations in discharge, which can cause rapid changes in water volume and associated variation in temperature and salinity (Warix et al. 2023). Such environmental variability, together with the drying process itself, can intensify environmental stress, reducing dissolved oxygen and ultimately causing desiccation (Bonada et al. 2020; Gómez et al. 2017). These conditions impose strong environmental filters on aquatic biota locally (Datry et al. 2014), while drying can enhance regional heterogeneity by favoring patchiness of environmental conditions among isolated pools, and reduce hydrological connectivity, limiting dispersal among communities (Escobar‐Camacho et al. 2025; Gauthier et al. 2020; Gianuca et al. 2024; Gonçalves‐Silva et al. 2025). Moreover, how the landscape surrounding river ecosystems is being used can influence aquatic macroinvertebrate communities. Typically, local and regional species diversity is reduced in urbanized and intensified agricultural regions compared to landscapes with a high percentage of natural cover (Gál et al. 2019; Schürings et al. 2022, 2024). Agricultural land use alters aquatic communities through increased chemical and nutrient loading, whereas urbanization promotes pollution and hydrological alterations, ultimately leading to the extinction of sensitive, narrow‐niched, geographically restricted, and less mobile species at multiple spatial scales (Shen et al. 2026). Therefore, it is urgent to integrate multiple ecological processes and biodiversity patterns to better understand how natural and anthropogenic stressors (e.g., drying, land use) affect freshwater biodiversity across scales.

The majority of river networks are composed of non‐perennial reaches and this proportion is expected to increase under global change in many regions (Abbasi et al. 2025; Döll and Schmied 2012; Messager et al. 2021; Mimeau et al. 2025). Although non‐perennial reaches typically exhibit lower local species richness (alpha diversity) (Datry et al. 2014; Soria et al. 2017), regional species richness (gamma diversity) may be maintained or even enhanced through increased differentiation in species composition among reaches (beta diversity), both spatially and temporally (Crabot, Mondy, et al. 2021; Datry et al. 2016; Escobar‐Camacho et al. 2025). These contrasting patterns are driven by dispersal limitation, high spatiotemporal environmental heterogeneity, local environmental filtering, and asynchronous community dynamics (Crabot, Mondy, et al. 2021; Escobar‐Camacho et al. 2025; Sarremejane et al. 2017). More broadly, these dynamics resonate with an ongoing debate on how biodiversity change manifests across spatial scales under global change, where stable local richness may occur alongside substantial compositional turnover (Dornelas et al. 2014; Gonzalez et al. 2016; Vellend et al. 2013). In this context, increases in beta diversity have been proposed as a potential mechanism buffering regional diversity; however, emerging evidence suggests that greater compositional differentiation does not necessarily compensate for local species loss nor ensure the maintenance of gamma diversity in terrestrial systems (Gonçalves‐Souza et al. 2025). Against this backdrop, it remains unclear whether increases in beta diversity in non‐perennial river systems can effectively compensate for local diversity loss and sustain gamma diversity, and whether this relationship is modulated by land use characteristics, particularly as expanding urbanization and intensifying agriculture may reduce environmental heterogeneity and promote biotic homogenization (Foley et al. 2005; McKinney 2006; Olden 2006), while simultaneously reducing landscape connectivity and constraining dispersal processes (Fahrig 2003). Consequently, the capacity of non‐perennial river networks to maintain regional biodiversity may depend on the balance between processes that generate compositional differentiation and those that promote biotic homogenization, determining whether these dynamic systems act as buffers or amplifiers of biodiversity loss in the Anthropocene.

The species‐area relationship (SAR) describes how the number of species increases with area or sampling units (Rosenzweig 1995), providing a powerful framework to integrate biodiversity patterns across spatial scales (e.g., Passy et al. 2023; Powell et al. 2013). Unlike traditional approaches that quantify alpha‐, beta‐, and gamma‐diversity at fixed and often arbitrary spatial scales, SAR explicitly captures how diversity components scale across sampling grains, allowing a continuous assessment of biodiversity patterns. When expressed on a logarithmic scale, the slope reflects the rate of species accumulation with area and is conceptually linked to beta‐diversity, whereas the intercept represents richness at the smallest scale and is analogous to alpha‐diversity (Scheiner 2003, 2004). Consequently, variation in SAR parameters across metacommunities can reveal scale‐dependent responses to global change stressors. For example, a negative effect on the intercept coupled with a positive effect on the slope suggests biodiversity loss at local scales that is offset by increased compositional variation among sites at broader scales (e.g., Powell et al. 2013).

**FIGURE 1:**
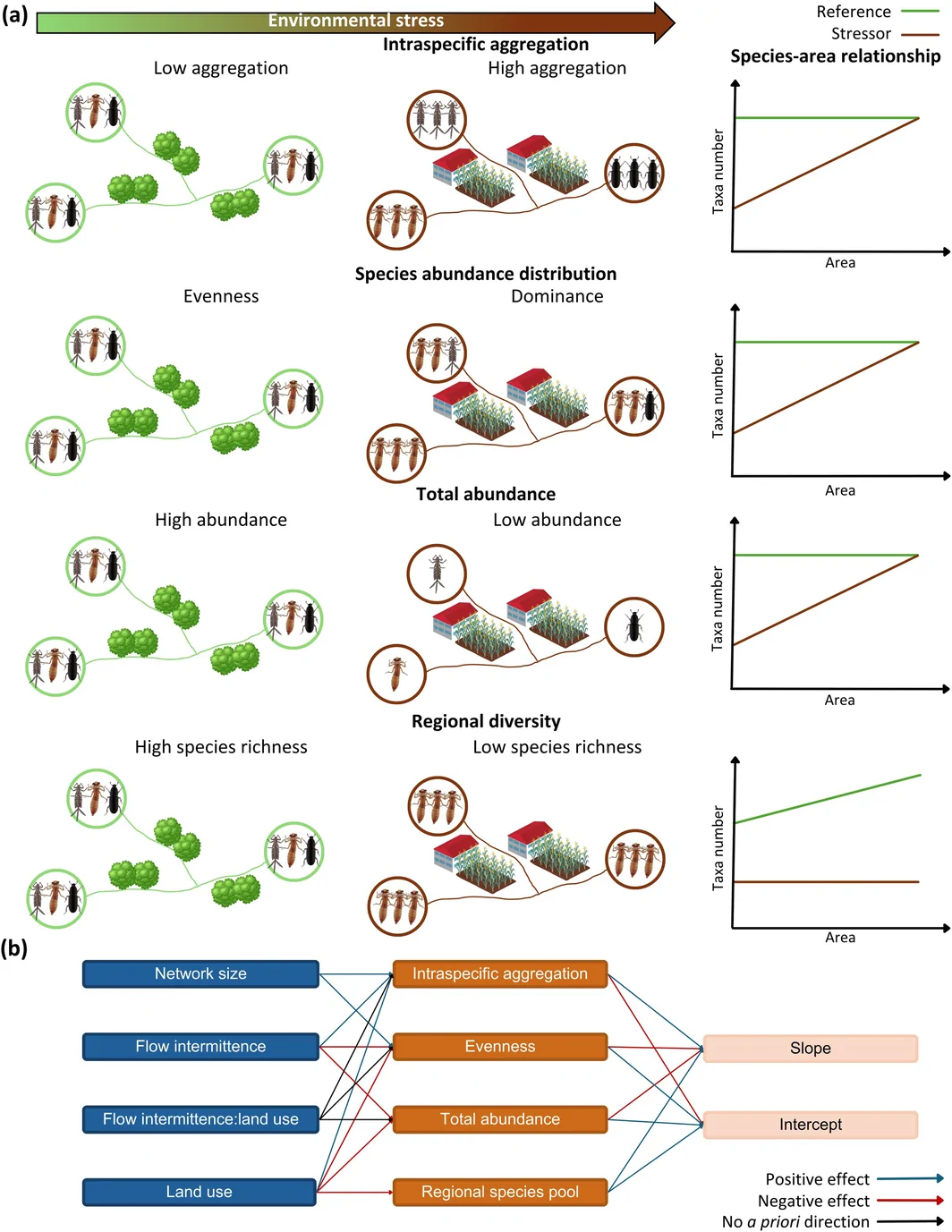
Conceptual model linking environmental drivers, metacommunity properties, and Species–Area Relationship (SAR) parameters (slope and intercept). (a) Conceptual representation of a river network illustrating the expected effects of flow intermittence and land use on four key metacommunity properties: Intraspecific aggregation, species abundance distribution, total abundance, and regional species pool. In each schematic, insect icons within circles represent different species distributed across sampling sites (local communities) within the river network (metacommunity). Green lines represent reference conditions (absence of the focal disturbance), whereas brown lines represent disturbed conditions (presence of flow intermittence or land use). Dotted brown river lines denote flow intermittence, whereas building and agriculture field icons denote land use. For each metacommunity property, the corresponding graph illustrates the expected consequences for the SAR, showing changes in species richness with increasing sampling area. (b) Hypothesized causal pathways linking network size, flow intermittence, land use, and their interaction to metacommunity properties and, ultimately, SAR parameters. Blue arrows indicate positive effects, red arrows indicate negative effects, and black arrows represent relationships that may emerge as positive or negative depending on interactions among predictors in shaping metacommunity properties (see main text).

Recent approaches move beyond simple descriptions of SAR parameters, linking species‐area patterns to metacommunity properties with key conservation implications (McGlinn et al. 2021; Passy et al. 2023). Importantly, SAR provides a unifying, scale‐explicit framework that integrates alpha, beta and gamma components, allowing direct inference of underlying metacommunity processes beyond traditional diversity partitioning. The main properties shaping SAR are: (1) intraspecific aggregation, (2) species abundance distribution (SAD), (3) total abundance, and (4) regional species pool size (Chase et al. 2018; He and Legendre 2002; McGlinn et al. 2019). Highly aggregated, uneven, and low‐density communities tend to have lower local diversity, lower intercepts, and steeper slopes, whereas smaller species pools yield both lower intercepts and shallower slopes since species are sampled more quickly across scales (Chase and Knight 2013; McGlinn et al. 2021) (Figure 1a). Although these properties clearly shape SARs, their responses to global change remain uncertain, particularly in river networks subject to variable flow intermittence. Flow intermittence may increase aggregation through dispersal limitation (Datry et al. 2016; Leibold et al. 2004), reduce abundance via drying (Asmamaw et al. 2021), and favor drought‐tolerant species through strong environmental filtering, leading to changes in SAD (Boersma et al. 2014; Vorste et al. 2016). Land use can further skew SAD, lower abundance, and increase aggregation by reducing habitat connectivity and by selecting tolerant species (Piano et al. 2020; Schürings et al. 2024). At the regional scale, gamma diversity may remain stable when declines in alpha‐diversity are offset by greater beta‐diversity (Escobar‐Camacho et al. 2025). However, habitat loss and pollution can also reduce beta‐diversity by homogenizing communities, potentially causing declines in gamma diversity. In addition, increasing distances among sites (i.e., network size) can constrain dispersal, thereby enhancing intraspecific aggregation (Shurin et al. 2009) and reducing dominance in metacommunity SADs by integrating a highly heterogeneous area. Two non‐exclusive outcomes may arise from the interaction between both stressors (i.e., drying and land use). First, they may reinforce the same dispersal‐ and environmental filtering mechanisms, producing synergistic effects. For example, both land use and flow intermittence may homogenize ecological communities through depletion of sensitive species or reduce connectivity between sites, thereby amplifying their effects on intraspecific aggregation and regional species dominance. Alternatively, the stressors may produce antagonistic effects, such that a non‐perennial metacommunity is less strongly affected by land use due to species co‐tolerance to both stressors (Soria et al. 2020; but see Di Cavalcanti et al. 2026, in production).

Here we used a large dataset of 49 metacommunities from rivers located in South‐Eastern France that vary considerably in flow intermittence and land use to (i) assess whether flow intermittence and land use influence local diversity and spatial turnover across communities, therefore reshaping the SAR curve, and (ii) examine how this influence is mediated by metacommunity properties such as intraspecific spatial aggregation, SAD, total abundance and regional species pool. Specifically, we hypothesize that flow intermittence would decrease the local diversity and increase the spatial turnover, with its effects on SAR parameters arising directly through impacts on metacommunity properties, following the framework of our theoretical model presented in Figure 1b. Conversely, we hypothesize that land use (i.e., urbanization and agriculture) will decrease both the local diversity and spatial turnover, therefore resulting in consistent biodiversity loss across all scales. We anticipate that our findings will advance understanding of scale‐dependent effects of flow regimes and land use on riverine biodiversity, and will provide actionable insights for conservation management in these environments.

## 2 Methods

### 2.1 Data Sampling

To develop this study, we used the dataset Naïades (http://www.naiades.eaufrance.fr/), which contains data from France rivers' monitoring. Surveys were carried out according to standardized protocols from Association Française de Normalisation for macroinvertebrates (AFNOR 2009), using Surber nets with 0.05 m^2^ of surface area and 500 μm of mesh. For each sampling site (i.e., local community), 12 sample points were defined. Since dispersal is a foundational principle of metacommunity dynamics (Leibold et al. 2004), we split the database between aquatic insects with aerial dispersion, and the others. We kept only the first because of the difference between the number of taxa registered (257 for insects, 70 for others) which could bias posterior analysis, and different taxonomic resolution, since all insect‐taxa were identified at sub‐family, family or genus level, while some non‐insect were identified only until Phylum or Classe, for example.

### 2.2 Metacommunities Description

Sampling sites were categorized and filtered as in Gianuca et al. (2024). For each sampling site, we assigned an intermittence level derived from Snelder et al. (2013). Snelder et al. (2013) modelled the probability of drying for each segment of the digital French river network, representing the likelihood that a given river reach exhibits intermittent flow based on long‐term flow records. We classified sites with flow‐intermittence probabilities of 0–0.15 as perennial and 0.25–0.85 as non‐perennial, thereby excluding sites with intermediate probabilities and reducing uncertainty in the flow‐regime classification.

Sites within the same hydrographic zone and sharing the same flow regime (perennial or non‐perennial) were then grouped into a single metacommunity. By restricting each metacommunity to a single flow‐regime category, we avoided mixing perennial and non‐perennial sites within the same metacommunity, which could obscure the interpretation of underlying processes and weaken our inferences. Hydrographic zones were defined using the BD CARTHAGE database (IGN), which delineates watersheds across the French territory based on topographic boundaries. This approach assumes that reaches within the same hydrographic zone constitute a metacommunity because they are potentially connected by hydrological pathways and dispersal processes. Although metacommunities were classified as non‐perennial or perennial, all further analyses treated flow intermittence as a continuous variable to account for the gradient in intermittence intensity among metacommunities. A detailed description of metacommunity delimitation is available in Gianuca et al. (2024).

For our analysis we selected only sites that were sampled for at least 6 years in a row (2009–2014) so we could have temporal replicates, and also metacommunities that contained a minimum of five local communities. Geographical visualization of data points is available at Figure S1.

To account for variation in the number of local sites per metacommunity (5–19), we generated all combinations of five sites within each metacommunity and computed parameter estimates for each combination, which were then averaged by metacommunity and year. When the number of possible combinations exceeded 1000, we randomly sampled 1000 combinations to ensure computational tractability. All subsequent metrics were calculated within this framework.

### 2.3 Land Use and Network Metrics

Land use data were obtained from the Copernicus Land Monitoring Service (CLMS) using the CORINE Land Cover 2018 set (https://land.copernicus.eu/en/products/corine‐land‐cover/clc2018) at a resolution of 100 m. The 44 original categories of land cover were simplified into four: urbanization, intensive agriculture and extensive agriculture, and natural land cover. For each local community, we computed the percentage occupied by each category inside a polygon drawn from the collection site to 1 km upstream and 500 m from riverbanks (Sponseller et al. 2001; Tormos et al. 2011; Wasson et al. 2010). As we were primarily interested in the overall effects of anthropogenic land use, urban and agricultural categories were combined into a single metric hereafter referred to simply as “land use”. Therefore, higher values of land use indicate landscapes that are more impacted by human pressures, whereas lower values indicate a higher percentage of natural land cover.

The spatial distance among sites within each metacommunity (i.e., network size) was calculated by first computing all pairwise distances among sites within each metacommunity using *st_network_cost* from the *sfnetworks* package (van der Meer et al. 2024). The minimum spanning tree connecting all five sites per combination was then derived based on river network spatial data from Snelder et al. (2013) using the *mstree* function of the *spdep* package (Pebesma and Bivand 2023). We considered only those combinations which could be completely connected along the river course. We ended up with 20 non‐perennial and 29 perennial metacommunities, sampled through 6 years, totaling 294 data rows.

### 2.4 Species‐Area Relationship

The SAR approach we applied is defined by Scheiner (2003) as a Type IIIB, where samples are taken from equally sized sites in a non‐contiguous grid. The formula used to fit the models was the log‐transformed power curve, given by $\log\left(S\right)=c+\log\left(A\right)\times z$, where c (intercept) and z (slope) are linear coefficients which links area or sample units A with richness S (Rosenzweig 1995). This was chosen since it is commonly the best fit and has a strong biological meaning (Passy et al. 2023; Scheiner 2003). Hereafter, sample units will be referred to as *area* for clarity.

For each combination of five sites, we fitted a species‐accumulation curve by *specaccum* function of *vegan* package (Oksanen et al. 2024), log‐transformed both species richness and the number of sample units, and fitted a simple linear model, extracting slope and intercept parameters.

### 2.5 Metacommunity Properties

The four key metacommunity properties were named and calculated as follows:

- Intraspecific spatial aggregation: hereafter referred to as *aggregation*, was measured as 1/k, where k is the dispersion parameter for all taxa in a metacommunity (Passy et al. 2023; Xu et al. 2015), estimated by maximizing the log‐likelihood function:

$$l=\sum_{i=1}^{\gamma }\;\left[o_{i}\;\log\left(p_{i}\right)+\left(m-o_{i}\right)\;\log\left(1-p_{i}\right)\right]$$

where γ is the metacommunity richness, m is the number of local species in the metacommunity, $o_{i}$ is the number of local communities occupied by species i, and $p_{i}$ is the predicted occupancy of species i, given by $p_{i}=1-{\left(1+\frac{n_{i}}{\mathrm{mk}}\right)}^{-k}$, being $n_{i}$ the abundance of species i;

II SAD: referred to as *evenness*, and measured as skewness of log‐transformed species abundances (*moments* package) (Komsta and Novomestky 2022), multiplied by −1 to simplify interpretation, such that the metric is proportional to equitability;
III Total number of individuals: referred to as *total N*, computed as the log‐transformed sum of all species abundances within the metacommunity; and
IV Regional species pool: referred to as *gamma diversity*, calculated as total species richness of metacommunity.

### 2.6 Species Traits

To assess how flow intermittence influences the direction of skewness in species abundance distributions (SADs), we used two trait classes linked to community stability: resistance (the ability to persist under disturbance) and resilience (the ability to recover after disturbance; *sensu* Lake 2013) represented by resistant forms and dispersal strategies, respectively. Resistance forms included the following modalities: none, housings against desiccation, eggs/stoblasts, diapause or dormancy, and cocoons, while dispersal strategies included aquatic passive, aquatic active, aerial passive, and aerial active modalities (Tachet et al. 2010). Species were assigned fractional degrees of membership to each trait modality through fuzzy coding, such that the memberships for all modalities of a given trait summed to one.

### 2.7 Statistical Analysis

We built structural equation models (SEM) based on our theoretical framework (Figure 1b) using the *piecewiseSEM* package (Lefcheck 2016). Each causal path was represented by a linear mixed‐effects model with sampling year and metacommunity identity included as random intercepts. Flow intermittence, land use, their interaction, and river network size were specified as exogenous predictors. A quadratic term for land use was also included in the gamma diversity model, as we detected a nonlinearity that improved the model's fit. Additionally, to better understand the effect of land use on our target system, we also fitted models separately for each anthropogenic land use category (urbanization, intensive agriculture, and extensive agriculture) (Figure S2–S4). Metacommunity properties (aggregation, evenness, total N, and gamma diversity) were included as endogenous mediators, whereas intercept and slope were treated as the ultimate response variables. All variables were standardized (*z*‐scores) to improve model convergence and facilitate comparison of effect sizes.

Overall SEM fit was assessed using Fisher's C statistic and the *χ* ^2^ test, with *p* > 0.05 indicating adequate fit. Because the initial SEM showed poor fit (Figure S5), we incorporated correlated errors among metacommunity properties and between the quadratic term of land use and the intercept. Model assumptions (residual normality and homoscedasticity) of single fitted models within the SEM were evaluated by visual inspection of residuals using the *performance* package (Lüdecke et al. 2021). Spatial autocorrelation of residuals was tested using Moran's I in the *DHARMa* package (Hartig 2024), with no evidence of significant autocorrelation (*p* > 0.05).

Direct, indirect, and total effects were estimated using 1000 bootstrap iterations with the *semEff* package (Murphy 2024), and 95% confidence intervals were computed. Because bootstrap estimates are not provided for correlated errors, these were obtained from the standard output of the *piecewiseSEM* summary.

As flow intermittence shifted species‐abundance distributions toward the dominance of a few species, we further investigated whether these changes selected for drought‐tolerant or more mobile taxa. We fitted generalized linear mixed models for each trait modality within dispersal and resistance trait classes. For each metacommunity, species abundances were summed and converted to relative abundances, which were then used to calculate community‐weighted means (CWM; Cardoso et al. 2025) for each trait modality. These CWMs, representing the relative dominance of each modality, were modeled using a beta distribution, with flow intermittence as a fixed effect and metacommunity identity and year as random intercepts. As we detected overdispersion, we also modeled an overdispersion parameter, within the *glmmTMB* package. To account for spatial autocorrelation found for “eggs, stoblasts” (M_obs_ = 0.234, M_exp_ = −0.021, *p* < 0.001), and “none” (M_obs_ = 0.190, M_exp_ = −0.021, *p* < 0.001) trait models, we computed distance‐based Moran's eigenvector maps (MEMs) using the *dbmem* function from the *adespatial* package (Dray et al. 2025). A forward, iterative selection procedure was implemented to account for spatial structure. At each iteration, MEMs were added individually to the model, and the eigenvector that most effectively reduced residual spatial autocorrelation (i.e., minimized Moran's I relative to its expected value) was retained. This process was repeated until residual spatial autocorrelation was no longer statistically supported (*p* > 0.05). To control for inflated Type I error rates arising from multiple related hypothesis tests, we adjusted *p*‐values using the Benjamini‐Yekutieli procedure (Benjamini and Yekutieli 2001).

Plots were generated using *ggplot2* (Wickham 2016), and SEM causal pathways were visualized with *DiagrammeR* (Iannone and Roy 2024). All analyses were conducted in R 4.5.1 (R Core Team 2025).

## 3 Results

### 3.1 Structural Equation Modeling

**FIGURE 2:**
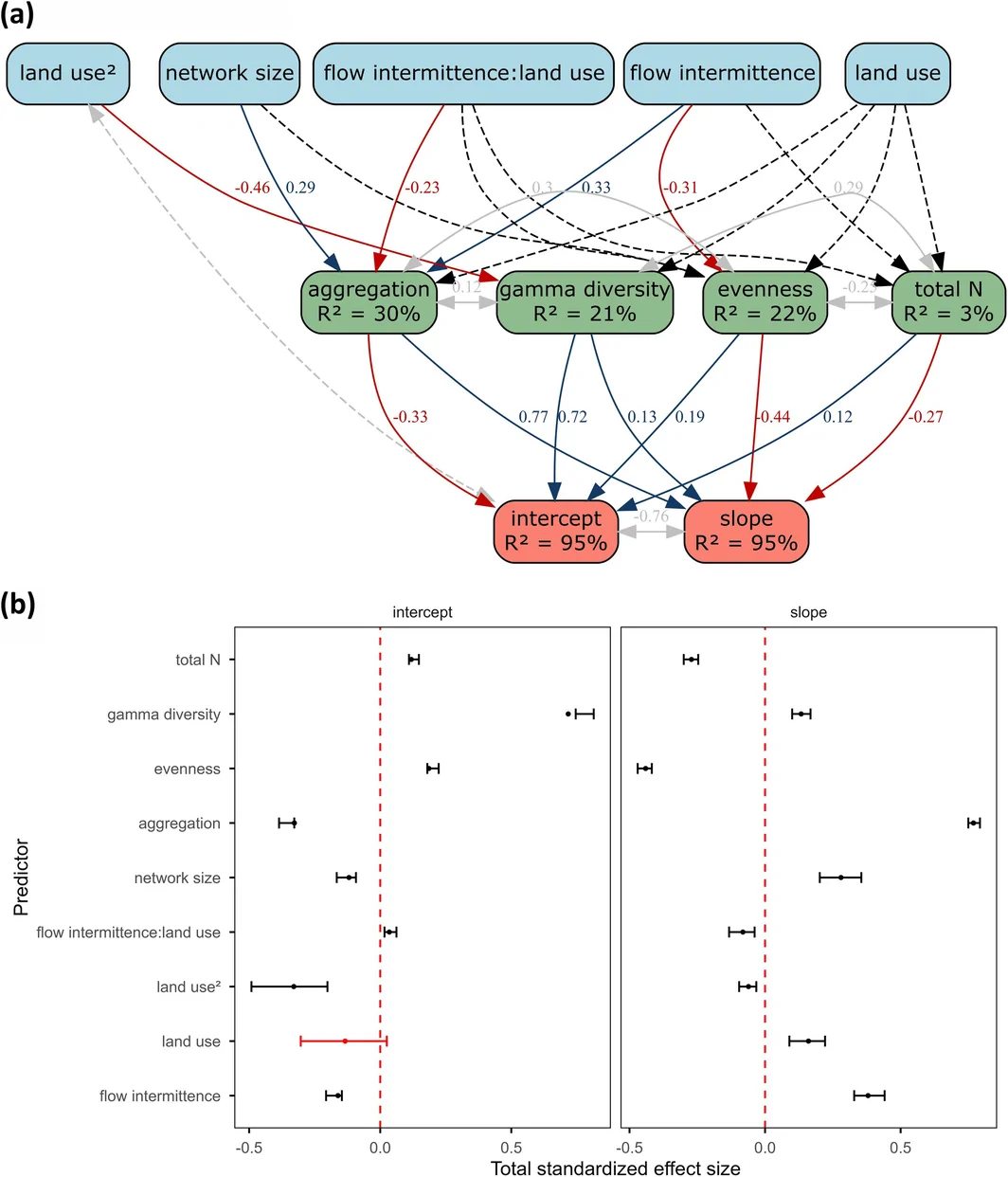
Structural equation model (SEM) results showing (a) direct pathways and (b) total (direct + indirect) standardized effects on the intercept and slope parameters of the SAR. In (a), red arrows indicate negative relationships and blue arrows indicate positive relationships, whereas double‐headed grey arrows represent correlated errors. Solid lines denote statistically supported paths (95% confidence intervals not overlapping zero), and dashed lines indicate unsupported paths. Numbers adjacent to arrows correspond to standardized path coefficients. In (b), points represent estimated effects and error bars denote 95% bootstrap confidence intervals. Interval confidences that cross the zero (dashed red line) are from non‐significant coefficients. Model fit statistics: *χ* ^2^ = 20.276, *p* = 0.208, df = 16; Fisher's C = 32.277, *p* = 0.355, df = 30.

Our SEM had a good fit based on both the chi‐square test (*χ* ^2^ = 20.276, *p* = 0.208, df = 16) and Fisher's C statistic (C = 32.277, *p* = 0.355, df = 30) after model refinement (Figure 2a). Flow intermittence increased aggregation, whereas its interaction with land use reduced aggregation (Figure S6), jointly explaining 30% of the marginal variance. Evenness was influenced solely by flow intermittence, which accounted for 22% of its variance. Gamma diversity was negatively affected by the quadratic term of land use, explaining 21% of its variance. Total N was not explained by any of the predictors. Both slope and intercept were strongly explained by metacommunity properties (95% variance explained in both cases). Indirect effects indicated that network size and flow intermittence increased slope and reduced the intercept, whereas land use exhibited a hump‐shaped (inverted U) relationship with both parameters (Figure 2b). The interaction between land use and flow intermittence positively affected the slope and reduced the intercept of the species‐area relationship. Splitting land use in different categories revealed that urbanization (Figure S2) negatively affected gamma diversity, whereas extensive agriculture (Figure S3) did not influence any response variables. In contrast, intensive agriculture (Figure S4) produced effects similar to those of overall land use, including a nonlinear relationship with gamma diversity and an antagonistic interaction with flow intermittence affecting aggregation.

### 3.2 Trait‐Based Environmental Filtering

Flow intermittence was associated with shifts in the relative dominance of dispersal modalities. Specifically, it increased the CWM of aerial passive dispersal (*β* = 0.352 ± 0.111, *z* = 3.18, *p*_BY (Benjamini‐Yekutieli)_ = 0.019) and decreased the CWM of aquatic active dispersal (*β* = −0.198 ± 0.060, *z* = −3.34, *p*_BY_ = 0.019). No significant effects were detected for aquatic passive (*β* = 0.041 ± 0.043, *z* = 0.943, *p*_BY_ = 1) or aerial active dispersal modalities (*β* = −0.149 ± 0.072, *z* = −2.08, *p*_BY_ = 0.189).

**FIGURE 3:**
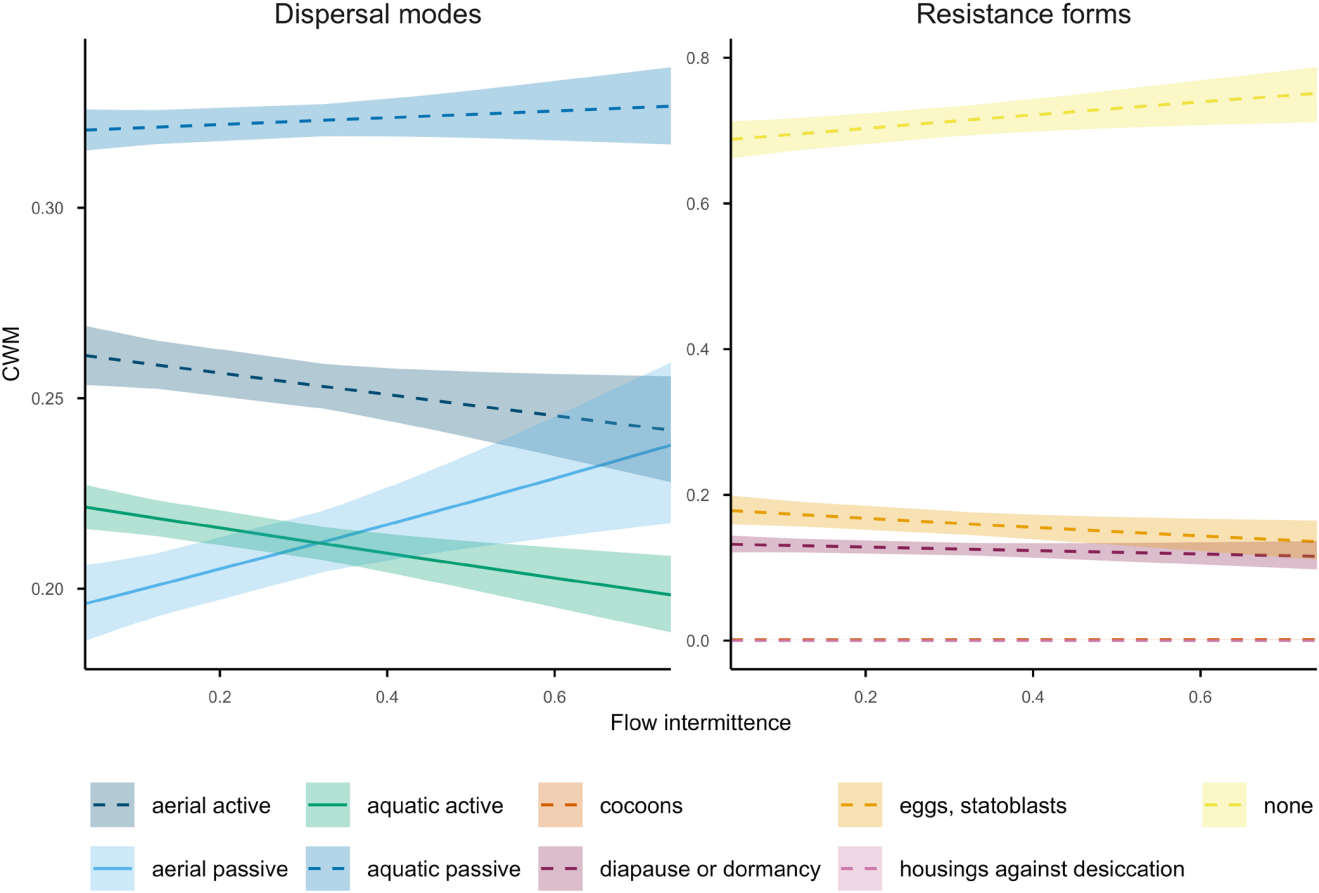
Effects of flow intermittence on the community‐weighted mean (CWM) of trait modalities associated with dispersal modes and resistance forms. Colors denote individual trait modalities (see legend), with cool colors representing dispersal modes (left panel) and warm colors representing resistance forms (right panel). Solid lines indicate significant relationships between flow intermittence and trait CWM (*p* ≤ 0.05), whereas dashed lines indicate non‐significant relationships (*p* > 0.05). Significance levels were corrected applying the Benjamini‐Yekutieli procedure.

For resistance traits, flow intermittence did not significantly affect the CWM of resistant eggs (*β* = −0.465 ± 0.203, *z* = −2.29, *p*_BY_ = 0.139), cocoons (*β* = 0.284 ± 0.236, *z* = 1.20, *P*_BY_ = 0.831), housings against desiccation (*β* = −0.285 ± 0.326, *z* = −0.875, *P*_BY_ = 1), diapause or dormancy (*β* = −0.219 ± 0.180, *z* = −1.21, *p*_BY_ = 0.831), or the absence of resistance forms (*β* = 0.452 ± 0.181, *z* = 2.50, *p*_BY_ = 0.107) (Figure 3).

## 4 Discussion

Our results demonstrate that increasing flow intermittence fundamentally reshapes species‐area relationships (SAR) in riverine metacommunities by reducing local‐scale diversity while simultaneously increasing spatial turnover across river reaches. These scale‐dependent responses emerged primarily through increases in intraspecific aggregation and shifts in species abundance distributions, whereas total abundance and regional species pools remained comparatively stable. In contrast, land use exerted a more pervasive effect across spatial scales, altering SAR parameters through nonlinear effects on gamma diversity and interacting with flow intermittence to modify patterns of species aggregation. Although mechanistic SAR frameworks have been increasingly applied to disentangle biodiversity scaling patterns in terrestrial systems (e.g., DeMalach et al. 2019; McGlinn et al. 2021; Paz et al. 2024; Powell et al. 2013), and more recently in perennial river networks (Passy et al. 2023), studies simultaneously addressing flow intermittence and land use in riverine ecosystems remain lacking. Here, by leveraging abundance data from hundreds of river reaches distributed across multiple metacommunities, we extend recent mechanistic SAR frameworks to non‐perennial river networks and demonstrate how flow intermittence, land use, and their interaction influence SARs through their effects on aggregation, species abundance distributions, and regional diversity properties. Together, these findings advance a mechanistic and scale‐explicit framework for understanding how global change reshapes biodiversity patterns in river networks.

### 4.1 Flow Intermittence Reshapes Scaling Parameters

Flow intermittence increased intraspecific aggregation in riverine metacommunities, which was expected due to flow interruption and therefore lack of connectivity between sites during dry seasons (Datry et al. 2016; Leibold et al. 2004). This relation between flow intermittence and aggregation can be explained by at least two non‐exclusive mechanisms. First, dispersal limitation is a known driver of spatial structure in metacommunities, as restricted movement of propagules prevents individuals from spreading uniformly and instead promotes recruitment near conspecific adults, leading to intraspecific aggregation (Condit et al. 2000; Nekola and White 1999). Such clustering is reinforced across generations through the dry season because colonization of distant river reaches is rare and local populations contribute disproportionately to nearby recruitment (Datry et al. 2017). Instraspecific aggregation has strong implications for the shape of the SAR because conspecific clustering reduces the likelihood of encountering many species in small samples, reducing the intercept of the SARs, while larger areas accumulate new conspecific clusters and thus additional species are sampled more rapidly, steepening the SAR slope (Grilli et al. 2012; He and Legendre 2002). Conversely, when dispersal rates are high, as would be expected in perennial rivers, individuals are more evenly distributed across space, reducing intraspecific aggregation and flattening SAR slopes. Together, theoretical models and empirical studies consistently demonstrate that limited dispersal leads to intraspecific aggregation, with steeper SARs, whereas high dispersal promotes spatial mixing and shallower slopes of SARs (Condit et al. 2000; Grilli et al. 2012; He and Legendre 2002).

The second mechanism linking flow intermittence to intraspecific aggregation is regional environmental heterogeneity, which is generally higher in non‐perennial than in perennial rivers (Snelder et al. 2013). Indeed, there is evidence that regional environmental heterogeneity can also generate intraspecific aggregation because species tend to establish and persist preferentially in subsets of the environment that match their ecological requirements (Chesson 2000; Leibold et al. 2004). Variation in water quality and microclimate across river reaches creates spatially structured environmental conditions and favors niche partitioning across the landscapes, leading conspecific individuals to be found clustered within patches of suitable habitat rather than evenly distributed across the landscape (Sarremejane et al. 2017). Consistent with this mechanism, a complementary a posteriori analysis showed that flow intermittence increases environmental overdispersion (Figure S7), suggesting greater heterogeneity of environmental conditions within non‐perennial metacommunities. This process is reinforced over time as recruitment success is higher within favorable microsites, leading to local demographic reinforcement of conspecific clusters (Harms et al. 2001). As with dispersal limitation, such environmentally driven aggregation shapes macroecological patterns: when suitable habitats are patchily distributed, as is the case for non‐perennial river reaches, increasing sampling areas encompasses a higher diversity of environmental conditions, exposing the survey to distinct habitat‐specific species assemblages and thereby fastly increasing the rate at which new species are discovered (Grilli et al. 2012; He and Legendre 2002). Consequently, environmental heterogeneity acts in concert with dispersal limitation to promote intraspecific aggregation and to shape the scaling of species richness across space.

Although previous studies have often emphasized the importance of resistance traits in the assembly process of non‐perennial river communities (e.g., Boersma et al. 2014; Vorste et al. 2016), our trait‐based analyses instead suggest that dispersal‐related filtering may play a stronger role in shaping species abundance distributions across non‐perennial river networks. Specifically, increasing flow intermittence favored taxa with aerial passive dispersal while reducing the relative dominance of aquatic active dispersers, indicating that drying may select against taxa whose dispersal is constrained by hydrological connectivity. Such shifts are consistent with metacommunity theory (Leibold et al. 2004) and suggest that flow interruption limits aquatic dispersal pathways and increases spatial isolation for essentially aquatic organisms among river reaches (Datry et al. 2016). In contrast, passive aerial dispersal can provide an alternative pathway for connecting isolated habitats, as wind‐mediated transport may facilitate movement across otherwise disconnected reaches without requiring individuals to actively locate and traverse the intervening landscape (Bilton et al. 2001). Although active aerial dispersers may have an advantage in directing movements towards suitable aquatic habitats using environmental cues (Bogan and Boersma 2012), passive dispersal may facilitate more stochastic and potentially long‐distance transport among isolated patches (Bohonak and Jenkins 2003; Fontaneto 2019). The greater representation of aerial passive dispersers under flow intermittence may therefore contribute to reduced evenness and increased dominance observed in non‐perennial metacommunities. Consequently, flow intermittence may reshape SAR scaling parameters not only through local environmental filtering, but also by reorganizing dispersal dynamics and spatial structuring across river networks.

Together, the simultaneous increase in intraspecific aggregation and dominance under increasing flow intermittence lowered SAR intercepts and steepened slopes, indicating reduced local diversity but greater compositional differentiation among river reaches (Crabot, Polášek, et al. 2021). Similarly, Gauthier et al. (2020) and Escobar‐Camacho et al. (2025) found that alpha diversity was lower and beta diversity higher in European non‐perennial rivers compared to perennial systems, although contrasting patterns may emerge in tropical regions. However, our study goes beyond demonstrating scale‐dependent biodiversity differences between perennial and non‐perennial rivers by explicitly revealing the metacommunity properties underlying these patterns. Specifically, our results suggest that changes in intraspecific aggregation and species abundance distributions can regionally compensate for local species loss by increasing compositional differentiation among river reaches. More broadly, these findings highlight the importance of integrating macroecological scaling approaches with metacommunity theory to understand how global change reshapes biodiversity patterns across spatial scales in dynamic river networks.

### 4.2 Nonlinear Effects of Land Use on Biodiversity

Land use altered SAR parameters primarily through its nonlinear effects on regional biodiversity. Gamma diversity (and ultimately SAR parameters) exhibited hump‐shaped responses, consistent with evidence that freshwater ecosystems often respond nonlinearly to landscape modification (Allan 2004). The initial increase in diversity likely reflects the greater environmental heterogeneity associated with moderately modified landscapes. Agricultural mosaics, for example, can vary in both compositional (e.g., crop diversity) and spatial (e.g., patch size, patch number, and ecotone length) configuration, creating a wider range of ecological conditions that may support additional species (Fahrig et al. 2011). In addition, these environments may facilitate the establishment of widespread or disturbance‐tolerant taxa without immediately causing the loss of habitat specialists, resulting in transient increases in diversity (Kuussaari et al. 2009; Tilman et al. 1994). Beyond a certain threshold of land use intensity, however, biodiversity declined and SARs became flatter, indicating reductions in both regional species pools and species accumulation across reaches. At these higher levels of disturbance, human‐modified landscapes generally simplify habitat structure, reduce environmental quality, and favor disturbance‐tolerant taxa at the expense of more specialized species (Foley et al. 2005; McKinney 2006; Schürings et al. 2022, 2024). Consequently, highly modified landscapes support smaller and more compositionally similar species pools, leading to lower SAR intercepts and flatter SAR slopes. This pattern is also consistent with the subsidy‐stress gradient hypothesis (Odum et al. 1979), whereby moderate habitat modification can enhance stream productivity through increased nutrient and organic matter inputs and greater light availability, benefiting aquatic invertebrate communities (Niyogi et al. 2007; Quinn 2000; Wagenhoff et al. 2011). At higher levels of disturbance, these same processes may become detrimental, causing ecosystem degradation through mechanisms such as eutrophication and algal blooms (Donohue et al. 2009; Quinn 2000).

The consistently negative linear effect of urbanization on gamma diversity further supports these interpretations on landscape heterogeneity and subsidy‐stress responses, highlighting that the nonlinear response observed for the overall land use gradient emerges from contrasting responses among its constituent categories. The absence of strong effects of anthropogenic land use on aggregation, species abundance distributions, and total abundance suggests that different forms and intensities of land use may impose distinct environmental pressures (Firmiano et al. 2021; Foley et al. 2005; Schürings et al. 2024), causing species losses while maintaining community structure among reaches.

### 4.3 Interaction Between Anthropogenic Stressors and Flow Intermittence

An important finding of our study was that anthropogenic impacts altered the effects of flow intermittence on metacommunity structure, particularly by reducing intraspecific aggregation within non‐perennial river networks. This result suggests that interacting global change drivers may reshape biodiversity scaling not only by intensifying local species loss, but also by modifying the spatial organization of communities across river networks. Flow intermittence alone likely promotes aggregation by increasing environmental heterogeneity and hydrological isolation among river reaches, thereby enhancing spatial differentiation among communities (Datry et al. 2016; Sarremejane et al. 2017). However, increasing land use may partially override these processes by homogenizing environmental conditions across the landscape, favoring widespread tolerant taxa and reducing compositional differentiation among reaches (McKinney 2006; Olden 2006). Consequently, although non‐perennial rivers may sustain high beta diversity under relatively natural and heterogeneous conditions, the combination of drying and anthropogenic land use may progressively erode this spatial differentiation, leading to more homogeneous metacommunity structure across river networks. More broadly, our findings reinforce the idea that multiple stressors can generate non‐additive biodiversity responses (Soria et al. 2020; Vinebrooke et al. 2004), highlighting that understanding biodiversity change in the Anthropocene requires explicitly integrating spatial scaling, metacommunity structure, and interacting global change drivers.

### 4.4 Implications for Non‐Perennial Rivers Management

Our findings highlight the importance of regional‐scale conservation strategies for non‐perennial river networks, as maintaining regional species pools is essential for preserving biodiversity and the ecosystem functioning and stability it supports (Gianuca et al. 2024). Such strategies should incorporate metrics that account for species compositional differences in biodiversity assessments and river‐basin management, rather than relying solely on local reach‐scale measures (Cid et al. 2022). Furthermore, the interaction between flow intermittence and land use reinforces the conclusions of Soria et al. (2020), although they focused only on small‐scale patterns, emphasizing the need for indicators capable of disentangling anthropogenic impacts from those associated with natural flow regimes. More broadly, understanding biodiversity dynamics and community assembly processes in non‐perennial rivers is becoming increasingly important, as these ecosystems are expected to become more widespread under ongoing global change (Abbasi et al. 2025; Döll and Schmied 2012). Consequently, naturally non‐perennial rivers may provide valuable insights into the ecological conditions and community responses likely to emerge in river systems that become artificially non‐perennial in the future.

## 5 Conclusion

Our results reveal that flow intermittence not only influences biodiversity patterns across scales in river networks but it also interacts with land use, completely altering metacommunity structure compared to what would be expected for perennial rivers. Specifically, non‐perennial rivers support lower local richness, but this loss is accompanied by greater compositional differentiation among reaches, showing that diversity can decline at local scales while being compensated at larger scales. Flow intermittence reshapes species‐area relationships mainly through changes in spatial aggregation and species abundance structure, whereas land use exerts threshold‐like effects that alter regional diversity and can weaken the spatial differentiation generated by drying regimes, leading to homogenization. Together, these findings show that global change drivers, including more prolonged and severe drought events and land use alterations, do not affect freshwater biodiversity uniformly: they redistribute diversity across scales, alter the balance between local loss and regional turnover, and can fundamentally change how communities are assembled across river networks. For non‐perennial rivers, conservation must therefore move beyond local richness and explicitly account for compositional heterogeneity, regional species pools, and the interacting effects of hydrological intermittence and land use intensification.

## Author Contributions

**Pedro Paulo Souza‐Lopes:** writing – original draft, conceptualization, formal analysis, software, visualization, methodology. **Thibault Datry:** writing – review and editing, funding acquisition, resources, data curation. **Carla Ferreira Rezende:** writing – review and editing, conceptualization. **Andros T. Gianuca:** writing – original draft, conceptualization, project administration, resources, supervision, writing – review and editing.

## Funding

This work was supported by Coordenação de Aperfeiçoamento de Pessoal de Nível Superior, 001. Conselho Nacional de Desenvolvimento Científico e Tecnológico, 409335/2023‐1. Agence de l'Eau Rhône Méditerranée Corse. Fundação Cearense de Apoio ao Desenvolvimento Científico e Tecnológico, ITR‐0214‐00226.01.00/26.

## Conflicts of Interest

The authors declare no conflicts of interest.

## Supporting information


**Figure S1:** Map of the study area in southeastern France. Colors indicate different metacommunities, defined according to hydrographic zones. Circles represent non‐perennial river reaches, whereas triangles represent perennial river reaches. The inset in the upper‐left corner shows the location of the study area (red rectangle) within an european context. A detailed description of metacommunity delimitation is provided by Gianuca et al. (2024), as referenced in the main text. Map lines delineate study areas and do not necessarily depict accepted national boundaries.
**Figure S2:** Structural equation model (SEM) accounting for urbanization as the land use metric. Red arrows indicate negative relationships and blue arrows indicate positive relationships, whereas double‐headed grey arrows represent correlated errors. Solid lines denote statistically supported paths (95% confidence intervals not overlapping zero), and dashed lines indicate unsupported paths. Numbers adjacent to arrows correspond to standardized path coefficients. Model fit statistics: *χ* ^2^ = 15.944, *p* = 0.386, df = 15; C = 26.590, *p* = 0.541; df = 28.
**Figure S3:** Structural equation model (SEM) accounting for extensive agriculture as the land use metric. Red arrows indicate negative relationships and blue arrows indicate positive relationships, whereas double‐headed grey arrows represent correlated errors. Solid lines denote statistically supported paths (95% confidence intervals not overlapping zero), and dashed lines indicate unsupported paths. Numbers adjacent to arrows correspond to standardized path coefficients. Model fit statistics: *χ* ^2^ = 19.510, *p* = 0.243, df = 16; C = 27.714, *p* = 0.586, df = 30.
**Figure S4:** Structural equation model (SEM) accounting for intensive agriculture as the land use metric. Red arrows indicate negative relationships and blue arrows indicate positive relationships, whereas double‐headed grey arrows represent correlated errors. Solid lines denote statistically supported paths (95% confidence intervals not overlapping zero), and dashed lines indicate unsupported paths. Numbers adjacent to arrows correspond to standardized path coefficients. Model fit statistics: *χ* ^2^ = 27.114, *p* = 0.167, df = 21; C = 43.111, *p* = 0.34, df = 40.
**Figure S5:** Initially fitted structural equation model (SEM) showing direct pathways between variables. Red arrows indicate negative relationships and blue arrows indicate positive relationships, whereas double‐headed grey arrows represent correlated errors. Solid lines denote statistically supported paths (95% confidence intervals not overlapping zero), and dashed lines indicate unsupported paths. Numbers adjacent to arrows correspond to standardized path coefficients. The model was not supported either by Chi‐squared or Fisher's C statistics. Model fit statistics: *χ* ^2^ = 84.152, *p* = 0, df = 21; Fisher's C = 107.189, *p* = 0, df = 40.
**Figure S6:** Conditional effects illustrating the interaction between flow intermittence and land use on intraspecific aggregation. Lines show model‐predicted aggregation values across the flow intermittence gradient for different levels of land use, with shaded areas indicating 95% confidence intervals. Green color represents less impacted metacommunities, while red color represents highly impacted metacommunities.
**Figure S7:** Effect of flow intermittence on within‐metacommunity environmental heterogeneity. Heterogeneity was measured as the multivariate distance of water parameters (temperature, pH, electrical conductivity, suspended matter, dissolved oxygen, DBO5, sulfates, nitrites, nitrates, hardness, phosphore, ortophosphates, chlorophylle a, and organic carbon) to the centroid. We fitted a generalized linear mixed‐effect model with Gamma family and log‐link, including flow intermittence as fixed predictor, and metacommunity identity and year as random intercepts. Although some observations appear visually extreme (grey dots), excluding them did not substantially alter parameter estimates or statistical significance. Results were consistent between the full model (β = 0.181 ± 0.039, z = 4.61, *p* < 0.001) and the reduced model (β = 0.160 ± 0.040, z = 4.046, *p* < 0.001).

## Data Availability

The data that support the findings of this study are openly available in Zenodo at https://zenodo.org/records/21180288.
