# Supplementary material for: Scale‐Dependent Biodiversity Responses to Drying and Land Use in River Metacommunities Facing Global Change
Source: Glob Chang Biol. 2026 Sep 4;32(9):e71094. doi: 10.1111/gcb.71094 (PMC13545490; doi:10.1111/gcb.71094)
Supplement: Supplementary file 1 [file GCB-32-e71094-s001.pdf]

Supplementary Material for

Scale-dependent biodiversity responses to drying and land use in river metacommunities facing global change

Pedro Paulo Souza-Lopes [1], Thibault Datry [2], Carla Ferreira Rezende [3], Andros Gianuca [4]

[1] Graduate Program in Ecology, Federal University of Rio Grande do Norte, Natal, Rio Grande do Norte, Brazil | [2] INRAE, UR Riverly, Centre de Lyon, Villeurbanne, Villeurbanne Cedex, France | [3] Postgraduate Program in Ecology and Natural Resources, Department of Biology, Federal University of Ceará, Fortaleza, Ceará, Brazil | [4] Department of Ecology, Federal University of Rio Grande do Norte, Natal, Rio Grande do Norte, Brazil

Correspondence: Andros Gianuca (andros.gianuca@ufrn.br, +55 (84) 99119-1561)

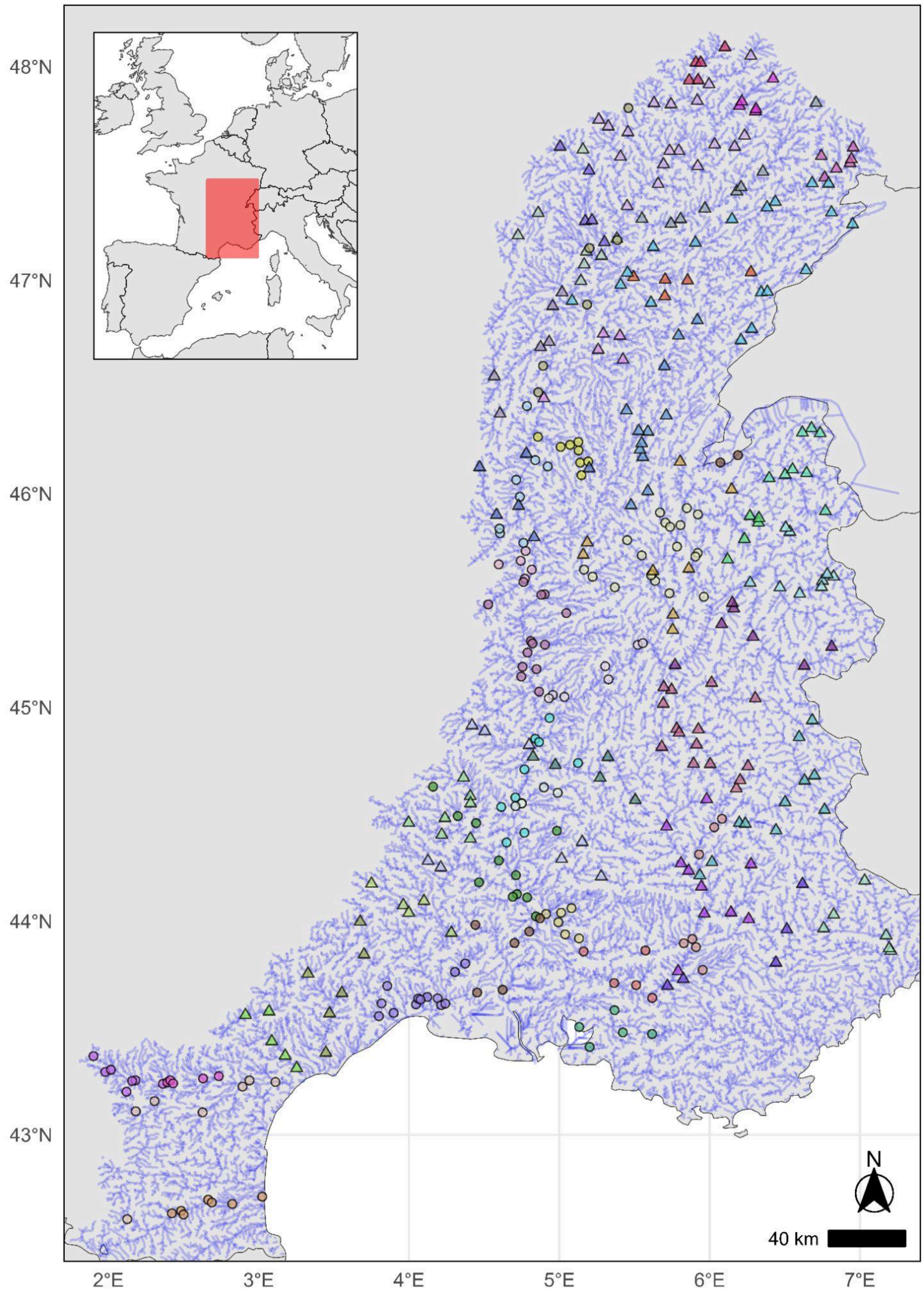

Figure S1. Map of the study area in southeastern France. Colors indicate different metacommunities, defined according to hydrographic zones. Circles represent non-perennial river reaches, whereas triangles represent perennial river reaches. The inset in the upper-left corner shows the location of the study area (red rectangle) within an european context. A

detailed description of metacommunity delimitation is provided by Gianuca et al. (2024), as referenced in the main text. Map lines delineate study areas and do not necessarily depict accepted national boundaries.

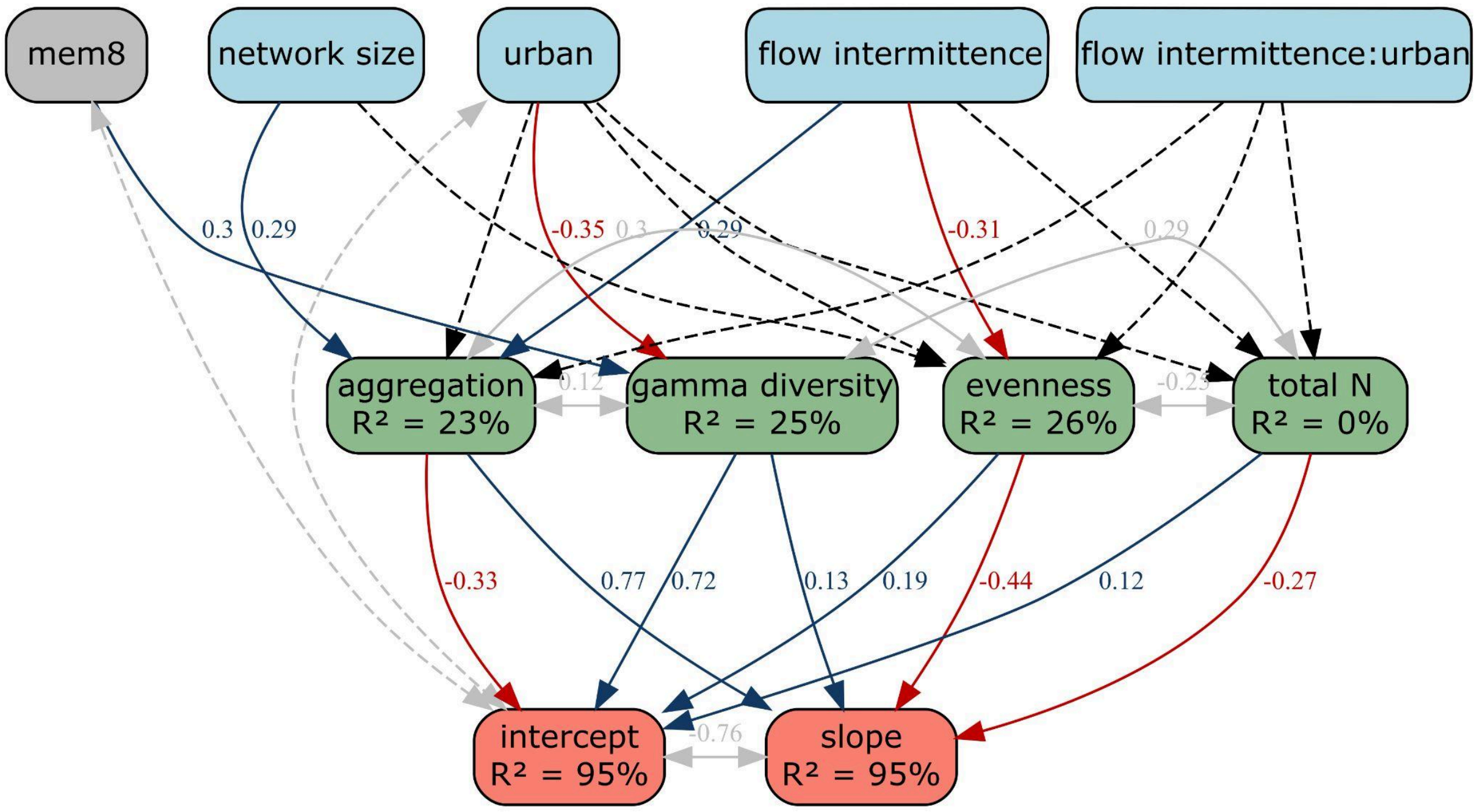

Figure S2. Structural equation model (SEM) accounting for urbanization as the land use metric. Red arrows indicate negative relationships and blue arrows indicate positive relationships, whereas double-headed grey arrows represent correlated errors. Solid lines denote statistically supported paths (95% confidence intervals not overlapping zero), and dashed lines indicate unsupported paths. Numbers adjacent to arrows correspond to standardized path coefficients. Model fit statistics: $X^2 = 15.944$, $p = 0.386$, $df = 15$; $C = 26.590$, $p = 0.541$; $df = 28$.

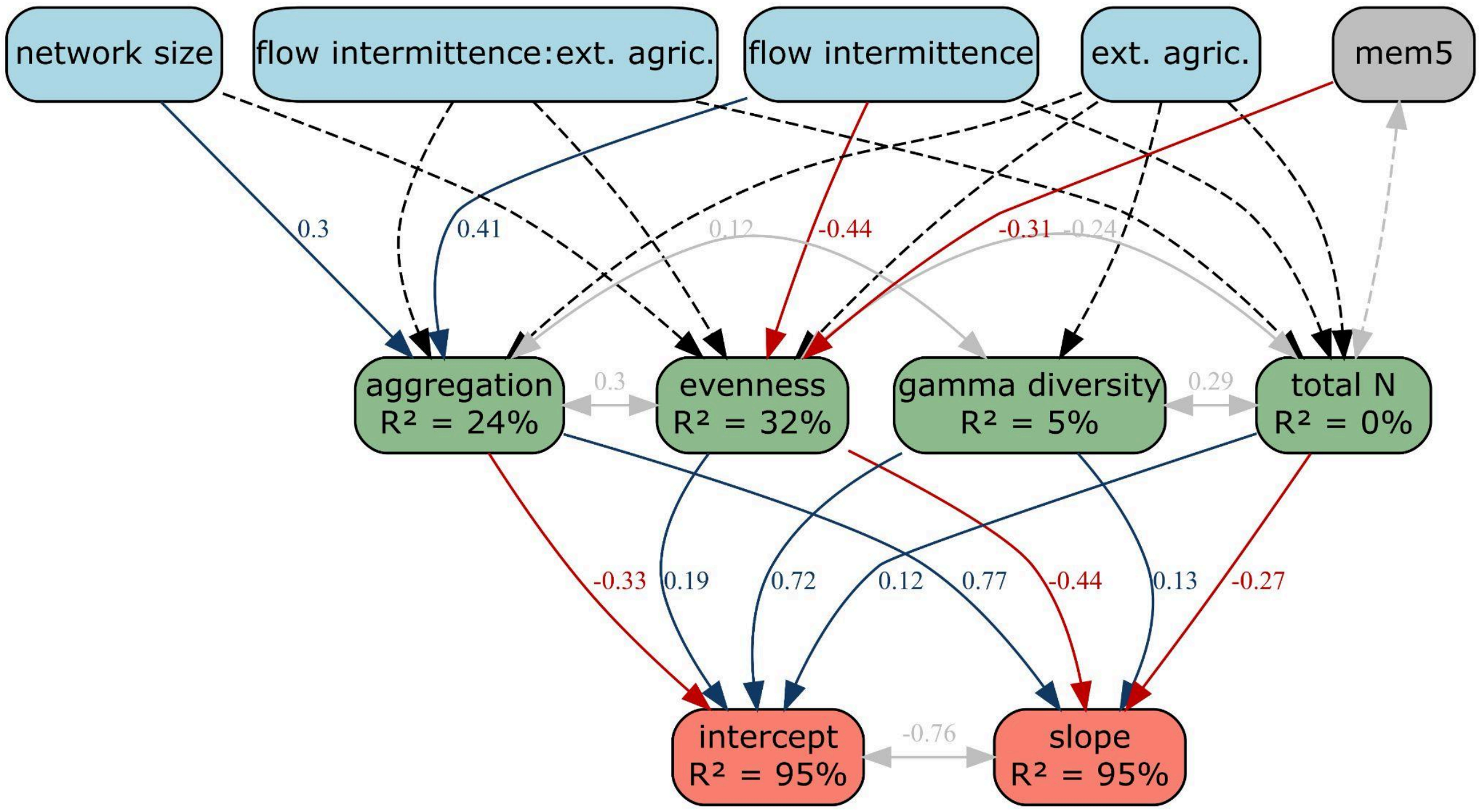

Figure S3. Structural equation model (SEM) accounting for extensive agriculture as the land use metric. Red arrows indicate negative relationships and blue arrows indicate positive relationships, whereas double-headed grey arrows represent correlated errors. Solid lines denote statistically supported paths (95% confidence intervals not overlapping zero), and dashed lines indicate unsupported paths. Numbers adjacent to arrows correspond to standardized path coefficients. Model fit statistics: $X^2 = 19.510$, $p = 0.243$, $df = 16$; $C = 27.714$, $p = 0.586$, $df = 30$.

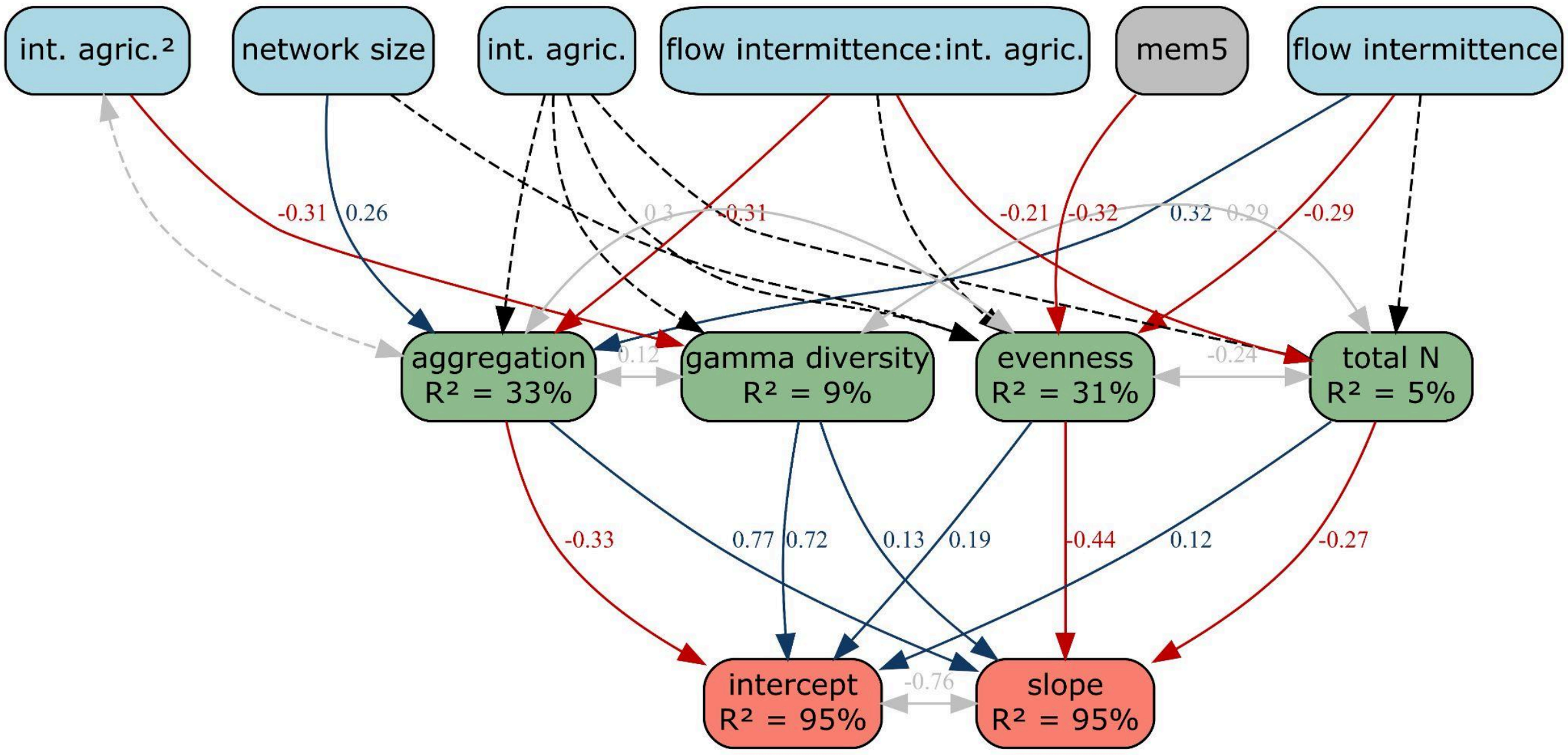

Figure S4. Structural equation model (SEM) accounting for intensive agriculture as the land use metric. Red arrows indicate negative relationships and blue arrows indicate positive relationships, whereas double-headed grey arrows represent correlated errors. Solid lines denote statistically supported paths (95% confidence intervals not overlapping zero), and dashed lines indicate unsupported paths. Numbers adjacent to arrows correspond to standardized path coefficients. Model fit statistics: $X^2 = 27.114$, $p = 0.167$, $df = 21$; $C = 43.111$, $p = 0.34$, $df = 40$.

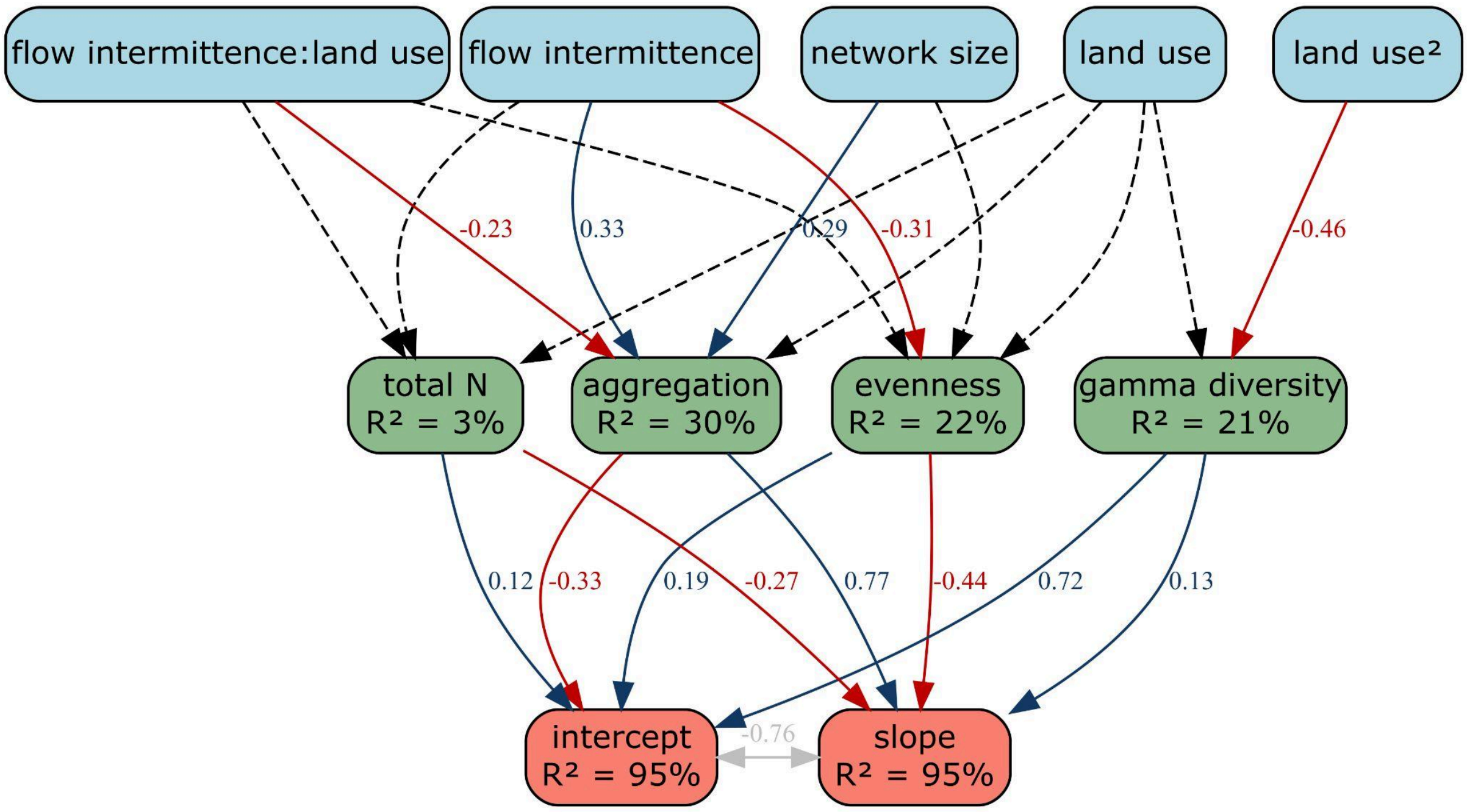

Figure S5. Initially fitted structural equation model (SEM) showing direct pathways between variables. Red arrows indicate negative relationships and blue arrows indicate positive relationships, whereas double-headed grey arrows represent correlated errors. Solid lines denote statistically supported paths (95% confidence intervals not overlapping zero), and dashed lines indicate unsupported paths. Numbers adjacent to arrows correspond to standardized path coefficients. The model was not supported either by Chi-squared or Fisher's C statistics. Model fit statistics: $X^2 = 84.152$, $p = 0$, $df = 21$; Fisher's $C = 107.189$, $p = 0$, $df = 40$.

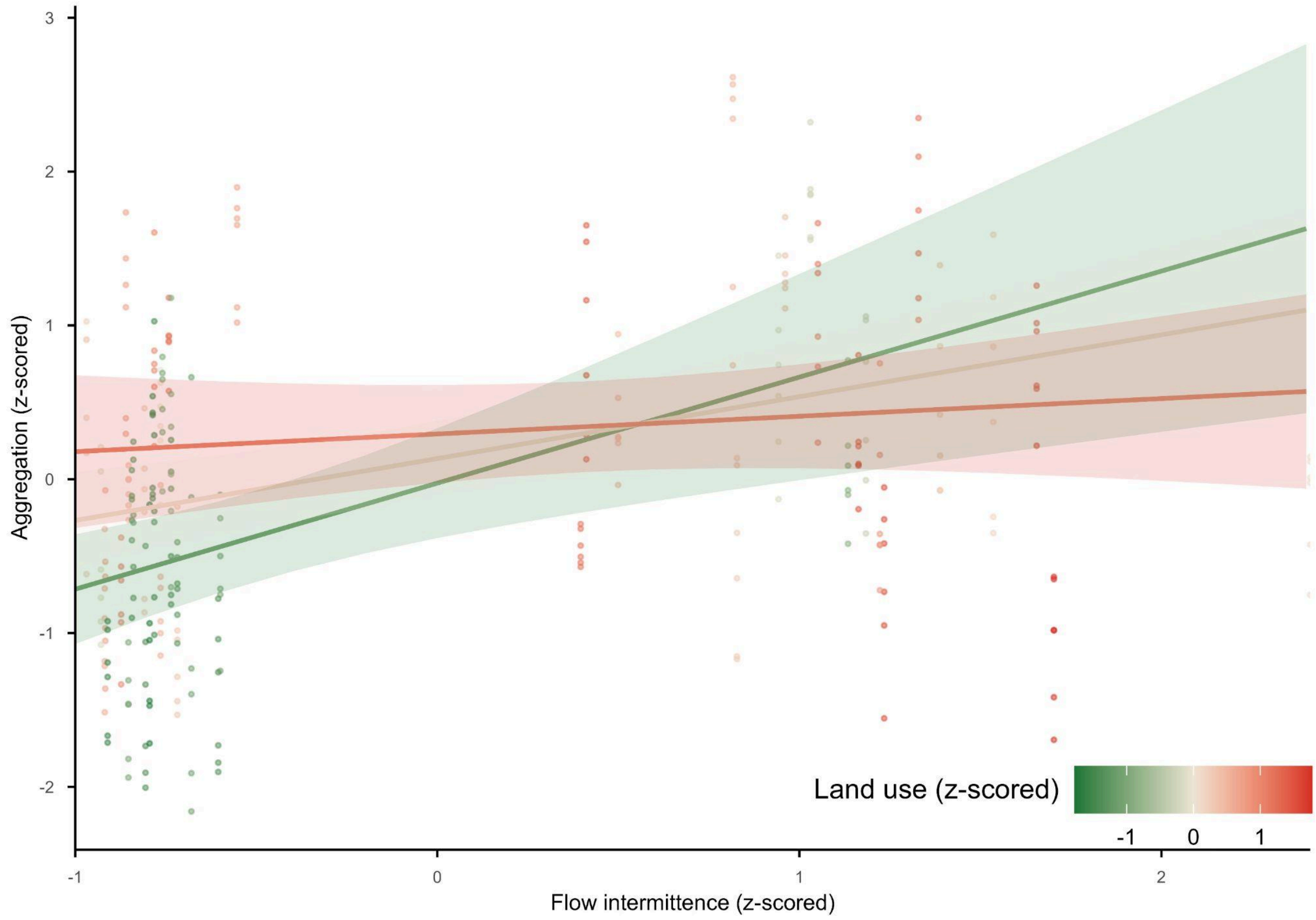

Figure S6. Conditional effects illustrating the interaction between flow intermittence and land use on intraspecific aggregation. Lines show model-predicted aggregation values across the flow intermittence gradient for different levels of land use, with shaded areas indicating 95% confidence intervals. Green color represents less impacted metacommunities, while red color represents highly impacted metacommunities.

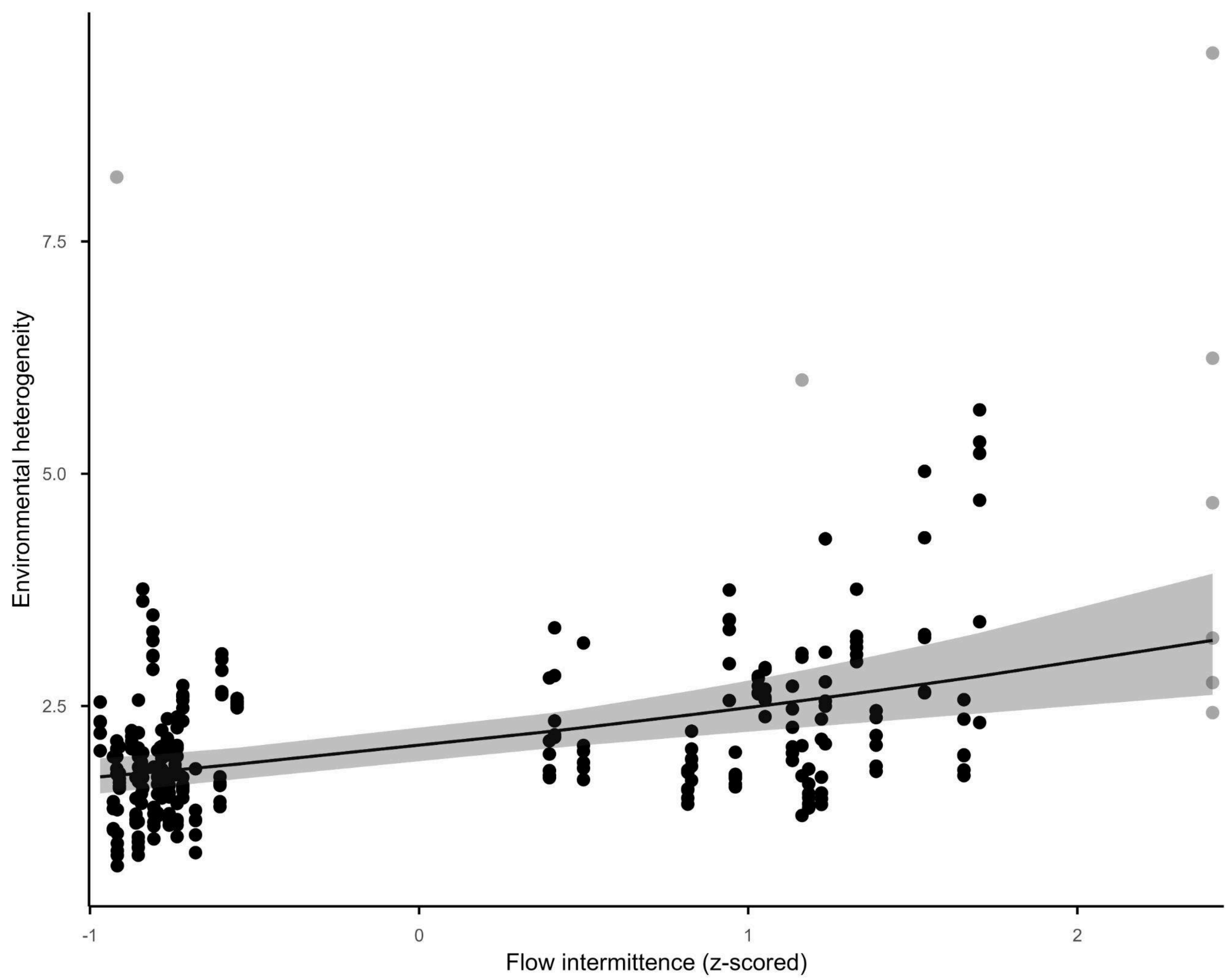

Figure S7. Effect of flow intermittence on within-metacommunity environmental heterogeneity. Heterogeneity was measured as the multivariate distance of water parameters (temperature, pH, electrical conductivity, suspended matter, dissolved oxygen, DBO5, sulfates, nitrites, nitrates, hardness, phosphore, ortophosphates, chlorophylle a, and organic carbon) to the centroid. We fitted a generalized linear mixed-effect model with Gamma family and log-link, including flow intermittence as fixed predictor, and metacommunity identity and year as random intercepts. Although some observations appear visually extreme (grey dots), excluding them did not substantially alter parameter estimates or statistical significance. Results were consistent between the full model ($\beta = 0.181 \pm 0.039$, $z = 4.61$, $p < 0.001$) and the reduced model ($\beta = 0.160 \pm 0.040$, $z = 4.046$, $p < 0.001$).
